# Crystal Structures of the Transcriptional Repressor RolR Reveals a Novel Recognition Mechanism between Inducer and Regulator

**DOI:** 10.1371/journal.pone.0019529

**Published:** 2011-05-03

**Authors:** De-Feng Li, Ning Zhang, Yan-Jie Hou, Yan Huang, Yonglin Hu, Ying Zhang, Shuang-Jiang Liu, Da-Cheng Wang

**Affiliations:** 1 National Laboratory of Biomacromolecules, Institute of Biophysics, Chinese Academy of Sciences, Beijing, People's Republic of China; 2 Graduate School of the Chinese Academy of Sciences, Beijing, People's Republic of China; 3 State Key Laboratory of Microbial Resources at Institute of Microbiology, Chinese Academy of Sciences, Beijing, People's Republic of China; University of Washington, United States of America

## Abstract

Many members of the TetR family control the transcription of genes involved in multidrug resistance and pathogenicity. RolR (***R*** **esorcin** ***ol*** ***R*** **egulator**), the recently reported TetR-type regulator for aromatic catabolism from *Corynebacterium glutamicum*, distinguishes itself by low sequence similarities and different regulation from the previously known members of the TetR family. Here we report the crystal structures of RolR in its effector-bound (with resorcinol) and aop- forms at 2.5 Å and 3.6 Å, respectively. The structure of resorcinol-RolR complex reveal that the hydrogen-bonded network mediated by the four-residue motif (Asp94- Arg145- Arg148- Asp149) with two water molecules and the hydrophobic interaction via five residues (Phe107, Leu111, Leu114, Leu142, and Phe172) are the key factors for the recognition and binding between the resorcinol and RolR molecules. The center-to-center separation of the recognition helices h3-h3′ is decreased upon effector-binding from 34.9 Å to 30.4 Å. This structural change results in that RolR was unsuitable for DNA binding. Those observations are distinct from that in other TetR members. Structure-based mutagenesis on RolR was carried out and the results confirmed the critical roles of the above mentioned residues for effector-binding specificity and affinity. Similar sequence searches and sequence alignments identified 29 RolR homologues from GenBank, and all the above mentioned residues are highly conserved in the homologues. Based on these structural and other functional investigations, it is proposed that RolR may represent a new subfamily of TetR proteins that are invovled in aromatic degradation and sharing common recognition mode as for RolR.

## Introduction

For survival in variable environments bacteria require a wide range of adaptive responses that are usually mediated by transcriptional regulators. Most microbial regulators known to-date are two domain proteins, namely a signal receiving domain and a DNA-binding domain[1]. Structural analyses have revealed that the helix-turn-helix (HTH) motif is a signature motif for the most recurrent DNA binding for prokaryotic transcriptional factors [2]. So far a series of prokaryotic transcriptional regulator families have been identified. Among them, the TetR family is well characterized and widely distributed in bacteria with an HTH DNA-binding motif [3]. The TetR family is named after its representative member, the TetR protein, which has been extensively characterized [3]. This TetR protein controls the expression of the *tetA/B/C* genes to confer resistance to tetracycline [4]. Members of the TetR family exhibit a high conservation of sequences for the DNA binding domain. Generally, proteins of TetR family are involved in the adaptation to complex and changing environments. So far only a few members of the family are characterized both functionally and structurally, including TetR [5], QacR [6], CprB [7] and EthR [8]. For all these proteins their effectors are rather large and involved in the complicated binding process with the conformational changes of the repressors, like the tetracycline for TetR and the multidrug efflux related-compounds for QacR. Here we report the crystal structures of a novel transcriptional repressor RolR and its complex with the regulator resorcinol.

RolR (***R*** **esorcin** ***ol*** ***R*** **egulator,** previously known as Ncgl1110) is a transcriptional repressor from *Corynebacterium glutamicum*. Further studies show that RolR belongs to the TetR family, and it regulates the resorcinol degradation in *C. glutamicum* [9]. RolR shows generally low sequence similarities to all structure-known TetR members, especially its C-terminal is completely different from all the known TetR-type regulators. Recently the resorcinol molecule has been identified as the effector of RolR (see [10] and supplemental material Figure S1). So far, resorcinol represents the simplest molecule compared to the currently known effectors for TetR-Type regulators. In addition, RolR is the sole TetR-type regulator that has been identified for regulation of aromatic catabolism. The structures of RolR and its complex with resorcinol show the unique regulator binding property and distinct structural elements for DNA binding domain to accommodate to small effectors like resorcinol. The structure-based mutagenesis analysis identified a unique recognition and binding mode between RolR and the regulator resorcinol. The homologous analysis reveals that RolR represents a novel subfamily of TetR proteins, which should be involved in aromatic degradation and sharing common recognition mode as for RolR.

## Results

### General structures of ligand bound- and free-RolR

**Figure 1:**
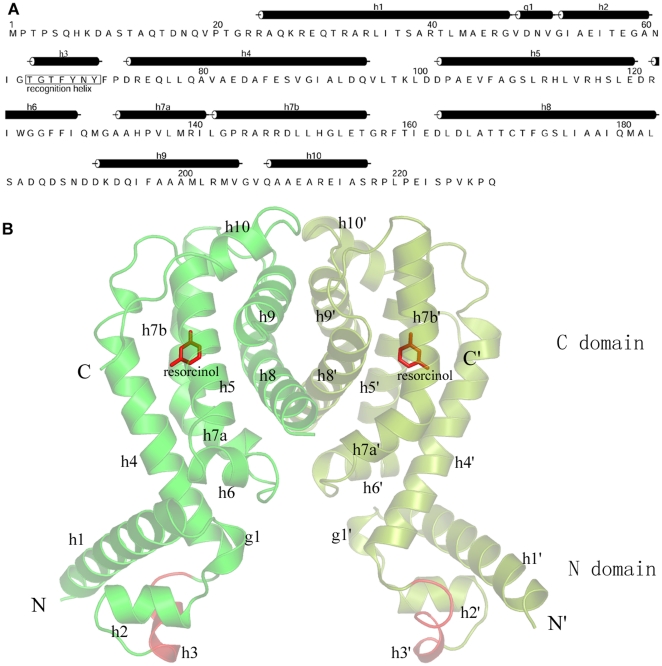
Overall structure of RolR. (a) Sequence and secondary structure distribution of RolR. (b) Ribbon presentation of the res-RolR dimer. The DNA binding helix (h3-h3′) is highlighted in red and the bound resorcinol molecules are shown in stick models (red).

The crystal structure of RolR complexed with resorcinol (res-RolR) has been determined using the MAD method at 2.5 Å resolution with R factor of 0.212 and R_free_ of 0.245. The statistics of data collection and structure refinement are shown in Table 1. The electron density maps are of good quality for fitting to the protein and the bound resorcinol. The structure shows a “Ω” like homo-dimer in the asymmetric unit (Fig. 1), which is similar to those observed in other TetR structures. Two subunits of the dimer, A and B, are generally identical to each other with a Cα r.m.s.d of 0.39 Å, so we will take subunit A as the representative in the following monomer-related analysis. RolR is an all-helix protein and each subunit is composed of 9 α helices (h1, h2, h4–h10) and two 3_10_ helices for h3 and g1, a stretch between h1 and h2 (Fig. 1). The helices are numbered as that commonly used in TetR proteins so as to facilitate the comparison. The RolR protomer is folded as two domains, the DNA-binding domain at N-terminal and the signal-receiving domain at C-terminal (Fig. 1).

**Table 1:** Data collection, phasing and structural refinement statistics.

	RolR-resorcinol complex			RolR
	SeMet peak	SeMet inflection	SeMet remoteH	
A. Data collection				
Wavelength (Å)	0.9795	0.9797	0.9644	1.0000
Space group	I23			P4_1_22
Cell constants				
a (Å)	167.12			152.95
b (Å)	167.12			152.95
c (Å)	167.12			117.48
α = β = γ (deg)	90			90
Resolution (Å)	50-2.50 (2.64-2.50)			108.46-3.60 (3.79-3.60)
Completeness (%)	100 (100)	100 (100)	100 (100)	98.1(93.8)
No. unique reflections	26929	26902	26814	16353
Redundancy	9.5	5.8	5.8	5.7
R_sym_ (%)	11.9 (49.7)	11.9 (49.9)	12.1 (50.2)	16.6 (50.1)
Average I/ 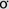	16.8 (3.5)	16.8 (3.2)	16.3 (3.3)	13.0 (2.9)
B. Phasing				
Selenium atom sites	10			
Resolution range of data used	50-3.0			
Overall figure of merit	0.64			
C. Refinement				
R (%)	21.2			22.4
R_free_ (%)	24.5			29.1
Protein atoms	3015			5690
Water molecules	16			0
Heteroatoms	159			0
Rms deviation from ideal				
Bond angels (deg.)	1.1			1.4
Bond lengths (Å)	0.006			0.014
Ramachandram analysis				
Most favored regions (%)	93.0			76.9
Allowed regions (%)	7.0			21.7
Generously allowed regions (%)	0			1.4
Disallowed regions (%)	0			0

Values in parentheses are for the highest resolution shell.

**Figure 2:**
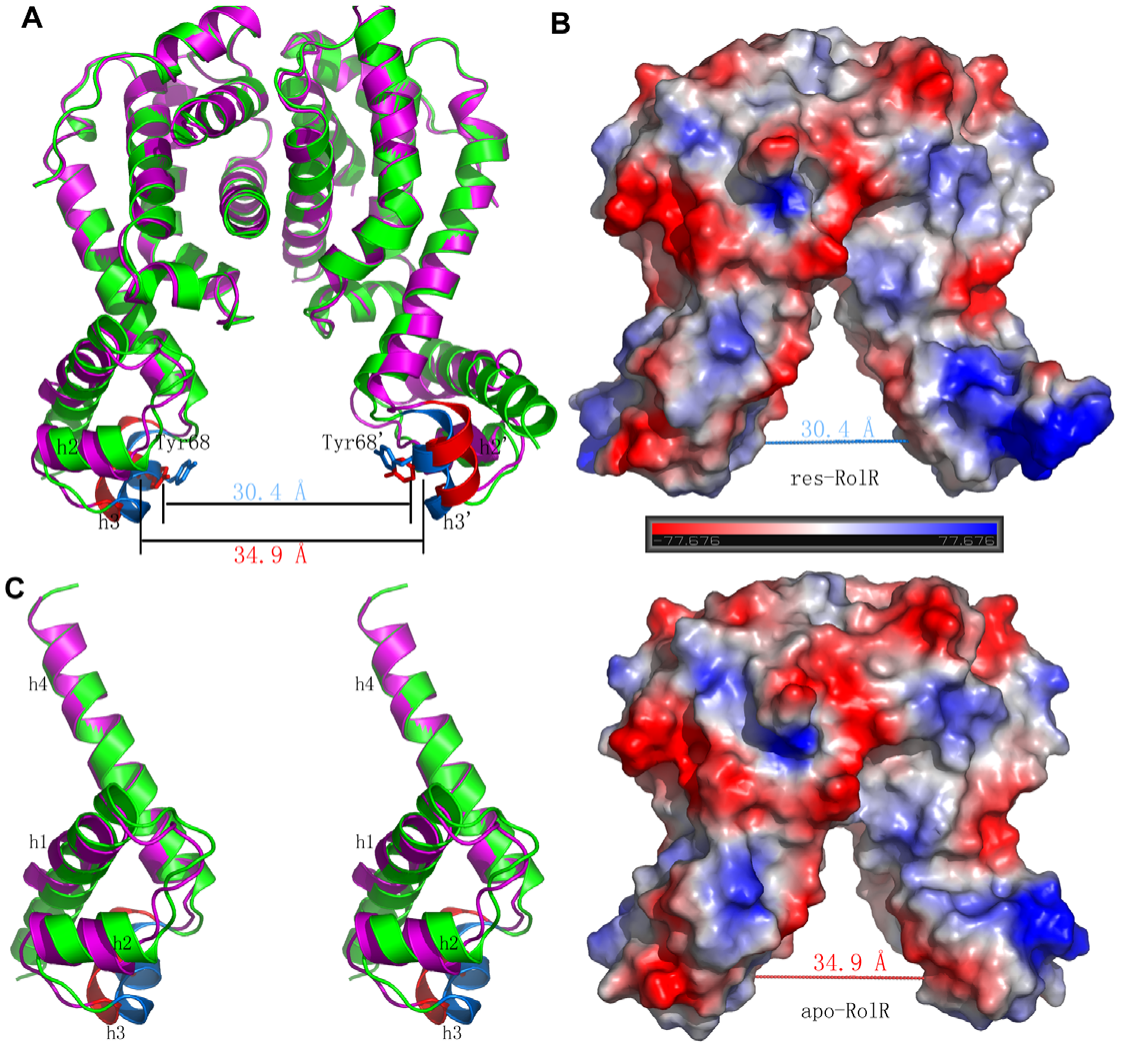
Comparison of ligand bound form (res-RolR, in green and its helix h3 in cyan) with ligand free form (apo-RolR, in magenta and its helix h3 in red) of RolR. (a) Superposition of whole molecule referring to the best fitting between C-domains. (b) Surface drawings of res-RolR (above) and apo-RolR (bottom) showing different separations of DNA binding domains. (c) N-domain (h1–h4) comparison of res-RolR and apo-RolR showing different orientations (about 10 degree) of the HTH DNA binding motif (h2–h3) in two RolR forms. Colors for res- and apo-RolR are same as in (a).

The crystal structure of the apo-RolR is solved by the molecular replacement method using the N- and C-domains of res-RolR structure as initial models. The structure is refined to 3.6 Å resolution and the statistics are listed in Table 1. At this resolution, the electron density maps are qualified for fitting well to all mainchains and about 1/3 sidechains, which make the reliability to analysis the apo-RolR structure on the mainchain and secondary structure level with some sidechains for a part of residues. The final apo-RolR model contains two dimers, AB and CD, in the asymmetric unit, which have the dimeric organization same as that in res-RolR. The general structures of these two dimers are very similar with a Cα r.m.s.d of 0.9 Å, we therefore use the dimer CD in the following comparative analysis due to its better fitting to density maps. The apo-RolR structure shows the similar general fold to that of res-RolR (Fig. 2a), but the DNA binding domain shows some relative movement in comparison with that in complex res-RolR (2a, 2c). Besides, some subtle distinctions for h3 and g1, the stretch between h1 and h2, are observed, which take a short 3_10_ helix and a loop, respectively.

### Unique binding mode between inducer and RolR

So far, the structure-known members of TetR family all bind to rather complex aromatic compounds, usually containing one more phenyl ring group, such as tetracyclines and quaternary ammonium compounds [3]. The structural analyses show that these TetR proteins recognize their inducer with an extensive multidrug-binding pocket and several conformational changes of the binding pockct-related parts [5], [6], [11]. As an effector of RolR, resorcinol is the simplest molecule compared to the currently know effectors for TetR-Type regulators. The resorcinol molecule only contains a phenyl group with two hydroxyl groups. The structure of RolR-resorcinol complex in comparison with the apo-form of RolR revealed unique recognition and binding properties that are distinct from the previously observed TetR proteins [3].

### 1) Binding pocket

**Figure 3:**
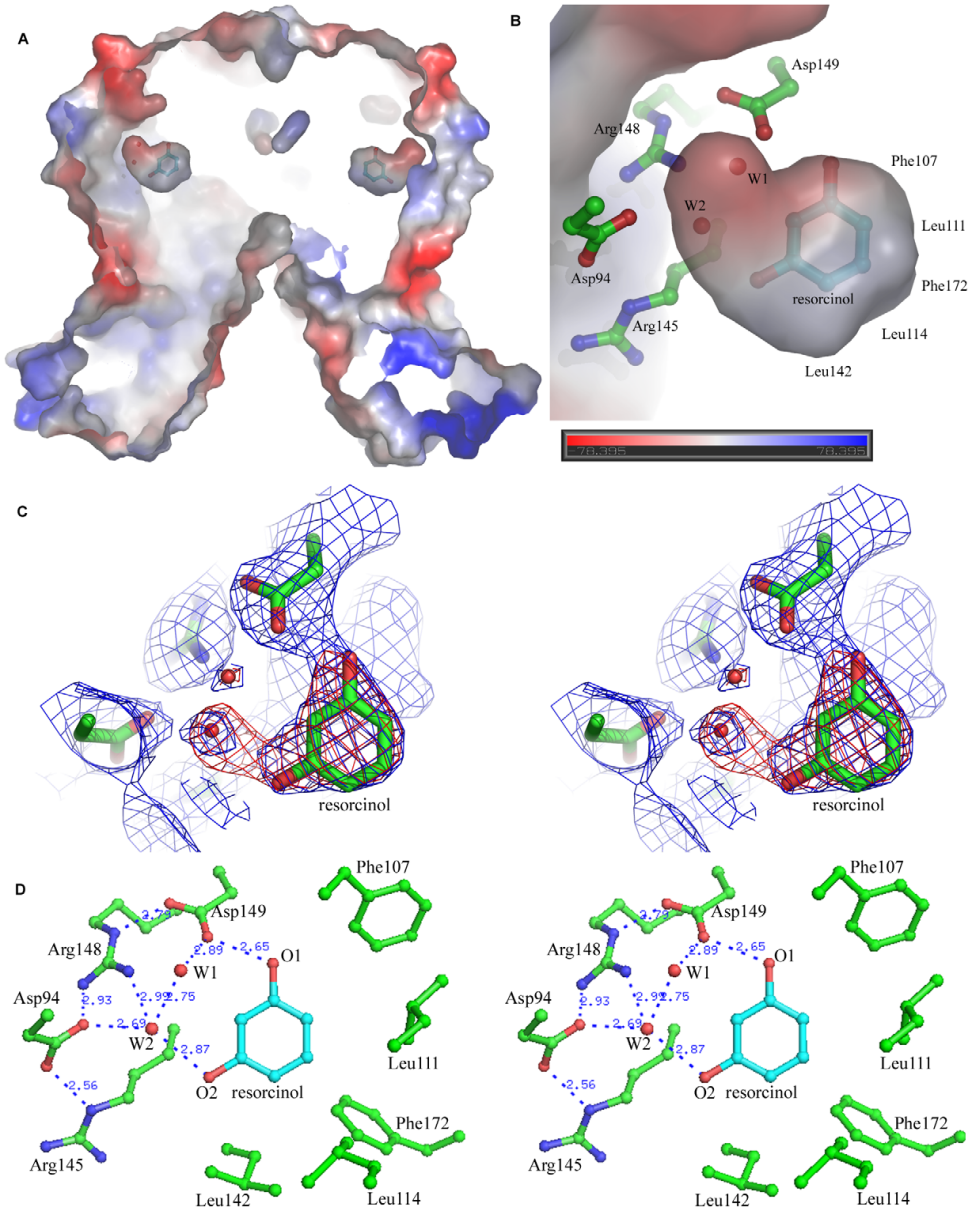
Recognition mode between RolR and resorcinol. (a)The electrostatic potential map of a longitudinal section drawing of res-RolR calculated with deletion of the resorcinol molecule showing the internal binding pocket between RolR and resorcinol. (b)The section around the resorcinol of the binding pocket fitting to a resorcinol molecule and two water molecules highlighted the water-mediated interactions between resorcinol and RolR. (c) The 2Fo-Fc (blue) and Fo-Fc (red) omit map around the bound resorcinol molecule, contoured at 1σ and 3σ respectively. (d) The recognition mode between RolR and resorcinol unique in a water molecules-mediated hydrogen bond network. The sidechains of residues involved in ligand recognition and water molecules are shown in ball-and-stick, with atoms O, N and C in red, blue and green, respectively.

Two resorcinol molecules are respectively bound to the two C-domains of the RolR dimer and the resorcinol-binding pockets are identical in both monomers. The binding pocket is formed by 5 helices (helices h4–h8) of the C-terminal domain (Fig. 1b, 3a). This pocket is a fully internal cavity. Structural comparison shows that the frameworks of the binding pockets in apo- and res-RolR structures are very similar without obvious conformational change at mainchains. This pocket is covered by the sidechains of residues Asp94, Arg145, Arg148 and Asp149 (Fig. 3b), the resorcinol molecule diffusing in or out of the binding pocket must be dependent on certain conformational changes of these residues' sidechains. The resorcinol binding pocket shows a volume about 250 Å^3^ as calculated by CASTp, which is the smallest one compared with those of other TetR members, ranging from 630 Å^3^ in SmeT to 1500 Å^3^ in TtgR [6], [7], [8], [12], [13], [14]. The cavity is much larger than the space for accommodation of one resorcinol molecule, which implies that RolR could also recognize some larger ligand molecules than resorcinol.

### 2) RolR-resorcinol recognition and binding mode

In the binding pocket, the resorcinol molecule is located in a internal amphiphilic cavity, in which a cluster of hydrophobic residues interacts with the phenyl ring and a hydrogen bond network is bound to atoms O1 and O2 of the resorcinol molecule (Fig. 3). This spatial arrangement restrains the overall freedom of the molecule, especially the orientations of both atoms O1 and O2 of resorcinol. A number of hydrophobic residues, including Phe107, Phe172, Leu111, Leu114 and Leu142 from helix h4–h8, are involved in contacting with the phenyl ring, while residues Asp94, Asp149, Arg145 and Arg148 interact with O1 and O2 atoms of resorcinol via a hydrogen bond network mediated by two water molecules, W1 and W2 (Fig. 3b, 3c). In this network residue Asp149 directly interacts with atom O1 of resorcinol, while residues Arg148 and Asp94 interact with atom O2 of resorcinol through water molecule W2. Interestingly, these two paths are closely connected each other via hydrogen bonds among residues Asp149, Arg148 and water molecules to form a unique recognition mode (Asp149-W1-W2-Arg148-Asp94) for binding with resorcinol. In addition, this recognition is further stabilized by a series of hydrogen bonds between W1 and Ser110, W2 and Asp94, Asp94 and Arg148, as well as Asp94 and Arg145 (Fig. 3d).

In fact, the water molecule-mediated recognition mode for resorcinol binding is frequently observed in proteins. For example, it is recently reported that the antitumor galectin AAL recognizes its bioactive ligand, the TF antigen, by using a Glu-Water-Arg-Water motif [15]. The peanut lectin employs the water bridges for generating carbohydrate specificity [16]. Diego et al., also reported that water molecules on the surface of the carbohydrate recognition domain of galectins were related to the galectins' affinity for carbohydrate ligand recognition [17]. It seems to be an effective strategy to take water molecules into the recognition mode to endow with certain flexibility and variability for ligand binding. This implies that RolR could bind some larger ligand molecules other than resorcinol with the similar binding mode as in res-RolR.

### 3) Roles of residues related to resorcinol binding identified by mutagenesis analysis

**Figure 4:**
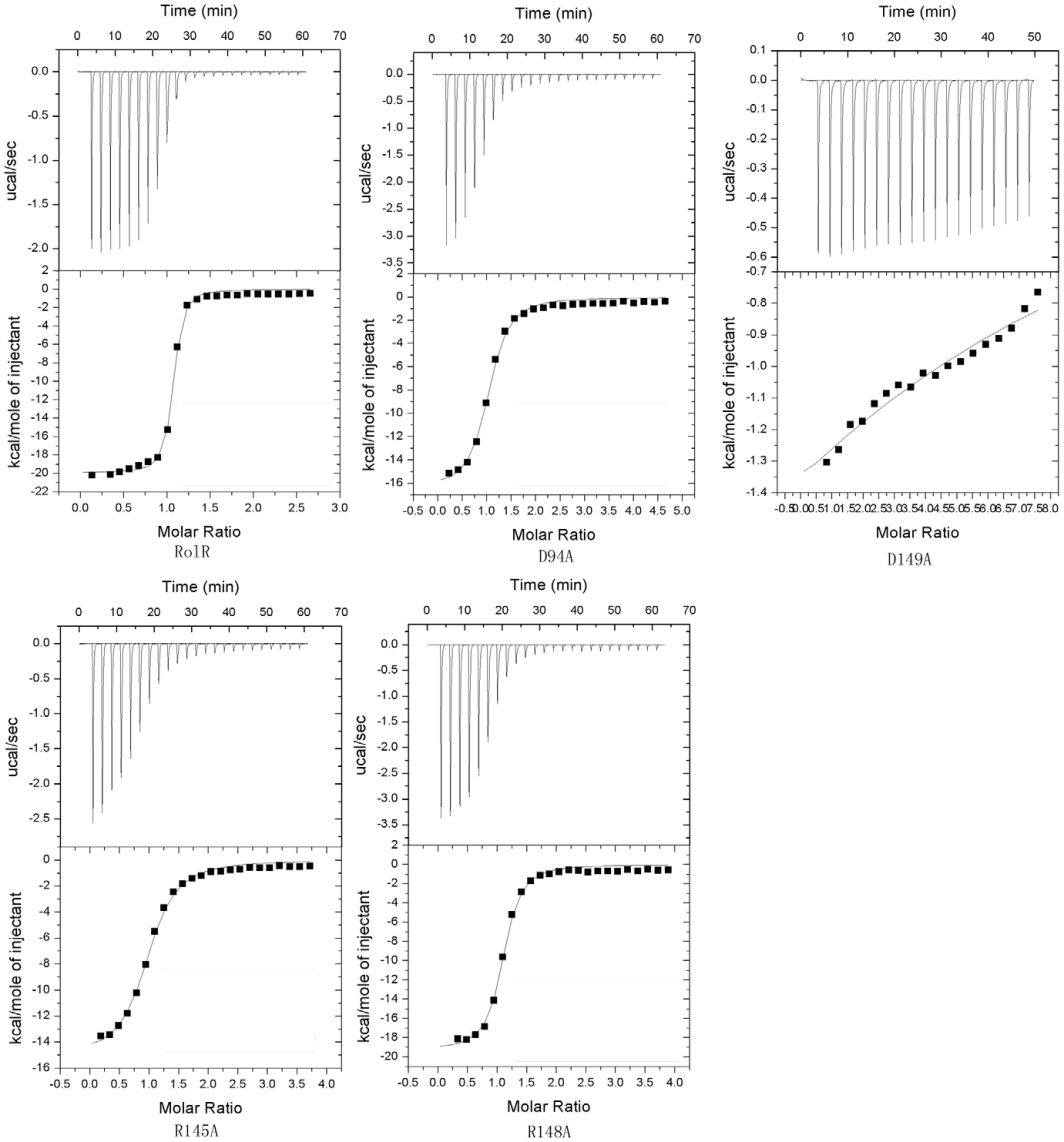
The plots of resorcinol titrating RolR and mutant proteins (D94A, R145A, R148A and D149A) using a MicroCal isothermal titration calorimeter. The results are summarized in Table 2.

To reveal the specific roles of the resorcinol-binding related residues in the hydrogen bond network, the mutations of D149A, R145A, D94A and R148A are constructed and expressed and these mutant proteins' performances of expression and purification are similar to that of native protein. The CD spectra of these proteins show all of them fold as native form (Figure S2) and the mutations do not disturb protein folding. Their affinities to the ligand resorcinol were analyzed by a isothermal titration calorimetry (ITC) respectively, in comparison with that of the wild type RolR (Fig. 4). The experimental data are fitted to a one-set-of-sites model and the resulted dissociation constants (Kd) are summarized in Table 2. The results show that the binding ability of mutant D149A to resorcinol is totally lost, while those of mutants R145A, D94A and R148A are dramatically reduced to 8.6%, 5.1% and 2.8%, respectively, in comparison with that of the wild type RolR (Table 2). The above data demonstrate that residue Asp149 is mainly involved in the specificity in the recognition of RolR with resorcinol ligand, while the residues Arg148, Asp94 and Arg145 are cooperatively involved in determination of the binding affinity of RolR to resorcinol. The water bridges in the hydrogen bond network should provide the certain flexibility for the recognition mode to accommodate the variant resorcinol-like inducers for different transcriptional regulatory effects.

**Table 2:** Summary of ITC experiment using resorcinol titrating RolR and mutant proteins.

	Kd (µM)	Relative affinity	n	ΔH (cal·mol^−1^)	ΔS (cal·mol^−1^·K^−1^)
RolR	0.19±0.03	100%	1.02±0.005	−1.990×10^4^±189.8	−36.0
D94A	3.7±0.51	5.1%	0.990±0.016	−1.648×10^4^±356.3	−30.4
R145A	6.7±0.71	2.8%	0.969±0.014	−1.509×10^4^±295.9	−26.9
R148A	2.2±0.29	8.6%	1.05±0.011	−1.926×10^4^±321.0	−38.6
D149A	-	0	-	-	-

The parameter for D149A binding resorcinol is too weak to be determined (Kd >1 mM).

### Distinctive structural properties of DNA binding domain

It is well known that most microbial regulators involved in the transcriptional regulation are two domain proteins with a signal-receiving domain (C-domain) and a DNA-binding domain (N-domain) transducing the signal. Structural analyses have identified that the helix-turn-helix (HTH) signature is the most recurrent DNA binding motif. In TetR family members, a couple of α helices, h2 and h3, in the dimeric organization constitute the shared HTH DNA-binding domain (h2–h3 and h2′–h3′) (Fig. 1, 2). The structural investigations of the TetR complexed with its signal molecule, tetracycline, in comparison with its DNA-binding form revealed that binding of the induce molecule would cause specific conformational changes in both the ligand binding pocket and the DNA-binding domain that result in release of the repressor from the operator, and thus allow transcription from the cognate promoter. For RolR the structural analysis shows some distinct structural properties of the DNA binding domain from that of known TetRs.

In the final structural models helix h3 critical for recognition with DNA adopts a 3_10_ helix in both res-RolR and apo-RolR calculated by programs Procheck and DSSP, which is different from that observed as an α helix in other TetR members. The segment h3 of RolR shows a glycines-rich (Gly63 and Gly65) sequence distinct from that in other TetR members, which may be the intrinsic factor for the relative conformational change of RolR. The structure of RolR shows some hydrogen bond interactions between the loop connecting h1 and h2 and the C-terminal domain (described in the following, e.g., residues Arg122 and Arg74), which restrain the orientation of helix h2 and, in turn, may help with the necessary stability of the 3_10_ helix. The structural comparison between apo-RolR and res-RolR show that the orientations of DNA-binding motif h2–h3 and h2′–h3′in dimerization have been swung about 10 degree referring to the C-domain from the ligand-free form to the ligand-binding form (Fig. 2), which further make the center-to-center separation of the DNA-binding domain (defined as the distance between residues Tyr68 in h3 and h3′ [8], [14]) shortened from 34.9 Å to 30.1 Å upon the ligand-binding (Fig. 2a, 2b). In case of other TetR members, such as TetR [5], [11], QacR [6], [18], [19] and EthR [8], the swing of N-domains are also observed as a common structural feature between ligand-bound and -free forms. It is therefore reasonable to believe that the N-domain swing observed in tow forms of RolR is intrinsic, but not from the different crystal packing. Accordingly, the peptide stretches (45–51) between h1 (32–44) to h2 (52–60) is transferred from a loop to a 3_10_ helix (48–51). This 3_10_ helix is stabilized by hydrogen bonds between N- and C-domains, i.e., hydrogen bonds Arg40^NH1^-Asp84^OD2^, Glu45^O^-Arg122^NH1^, Arg46^O^-Arg122^NH2^, Val51^O^-Arg74^NH2^, and one mediated by a water molecule, Val48^N^-water-Arg122^O^. In this way, residues Arg122 and Arg74 restrain the conformations of residues Glu45, Arg46, Val48 and Val51 via hydrogen bonds in the ligand-bound form but not in the ligand-free form after the 10 degree swing. It indicates that the DNA-binding domain is rather rigid in res-RolR comparing with that in apo-RolR which should be unsuitable for DNA binding.

The observations show a rather special structural properties of the DNA binding domain, in which the recognition helix h3 adopts a 3_10_ helix and the center-to-center separation between recognition helices h3 and h3′ is reduced from 34.9 Å to 30.4 Å upon inducer binding. In all TetR members structure-known to date the recognition helix h3 takes α-helix type conformation and the corresponding center-to-center distance is otherwise increased upon inducer binding, e.g. from about 34 Å to 39 Å, 41 Å and 52 Å for TetR [5], [11], QacR [6], [18], [19] and EthR [8], respectively. It seems that the structural change of RolR during DNA binding and release may represent a special model for the TetR-type transcriptional regulators.

## Discussion

**Figure 5:**
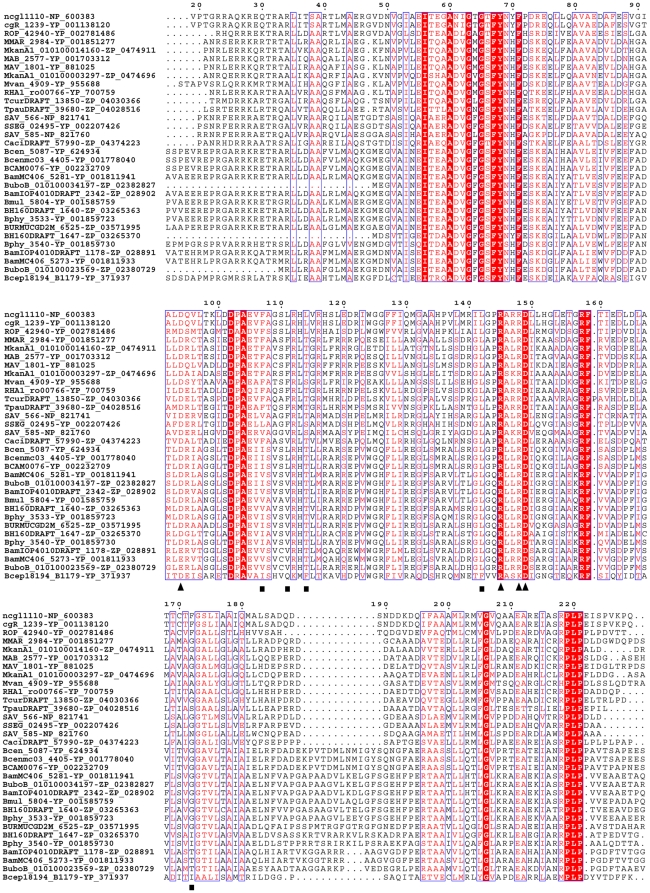
The sequence alignment of RolR (ncgl1110) and its similarities. The highly conserved residues (Asp94, Arg145, Arg148 and Asp149) involved in recognition mode and the hydrophobic residues (Phe107, Leu111, Leu114, Leu142 and Phe172) contacted with resorcinol molecule are highlighted by triangles and squares, respectively.

The structure of res-RolR complexed with the inducer resorcinol reveals a distinctive inducer recognition mode unique in a hydrogen bond network based on a water molecule mediated tetra-residues motif (Asp149-Water1-Water2-Arg148-Asp94-Arg145) (Fig. 3) and a hydrophobic cluster including Phe107, Leu111, Leu114, Leu142 and Phe172. Sequence similarity search based on the C-domain of RolR using BLAST reveals 29 proteins to be homologues with RolR in the non-redundant protein sequence database with except value less than 10^−4^, which all belong to TetR family. Most of these sequences are not functionally identified so far. The further sequence alignments with full length of the proteins to that of RolR show that the four residues involved in the inducer recognition mode of RolR are all identical (Asp149) or highly conservative (Arg145, Arg148, Asp94) (Fig. 5). In addition, the hydrophobic residues, including Phe107, Phe172, Leu111, Leu114 and Leu142, involved in the ligand binding pocket to contact with the aromatic ring of the resorcinol molecule, are also conserved as hydrophobic residues in the homologues (Fig. 5). The observations indicate that these transcriptional repressors should commonly adopt the unique recognition mode as observed in RolR to interact with corresponding regulators, which may take certain resorcinol-like compounds as their effector molecules.

For RolR, the DNA binding domain is also distinct from that observed in other TetR members. The DNA-recognizing helix h3 adopts the 3_10_ type in RolR, while the α type in other TetR members [5], [6], [7], [8]. In addition, the main apparent conformational change of the DNA binding domain induced by effector binding is the variability of the separation between DNA binding motifs (h2–h3 and h2′–h3′), which is shortened from 34.9 Å to 30.1 Å upon the inducer binding, while increased from about 34 Å to 39–50 Å in other TetR members identified to date [5], [6], [8], [11], [18], [19]. The sequence alignment shows that the DNA-binding motif h2–h3 characteristically possess a consensus sequence motif, _56_IxxxAxxGxGxFYxxF_71_ in RolR and all homologues (Fig. 5), which is distinct from that in other TetR members. It implies that RolR and its homologues may have the similar structural properties for DNA binding domain in the induced and non-induced status.

The observations and insight of RolR structures reveal a unique inducer-regulator recognition and binding mode and special structural change of the DNA binding domain. Based on the unique mechanism of RolR and its effector interaction, supposed sequence homologues of 29 proteins in alinment include both orthologs and paralogs, it is proposed that RolR may represent a novel subfamily of TetR proteins. Member of this subfamily should have the following features: 1) taking resorcinol-like molecules as effector and causing a reduction of space at the effector-binding demain upon the effector-regulator binding; 2) Unique in a hydrogen-bonded network and a hydrophobic region for the effector-binding domain of the regulator ; and 3) function as a transcriptional repressor in bacterial catabolism for resernal-like aromatic compound degradations. Certainly, the above speculations need further investigations on some members of the proposed subfamily to be identified.

## Materials and Methods

### Clone, Expression, Purification and Crystallization

The RolR gene was cloned on the pET-28a (Novagen) vector plus a His-tag by PCR with NdeI and HindIII restricted enzyme sites. The recombinant plasmid was transformed into *Escherichia coli* strain BL21(DE3) for expression. The overexpressed protein was purified by Ni–NTA affinity chromatography and size-exclusion chromatography. The purified protein was concentrated to about 15 mg/ml for crystallization. All crystallization experiments were performed with the hanging-drop vapor-diffusion method at room temperature. The drops were formed by mixing 1 µl of protein solution with 1 µl of reservoir solution and equilibrated against 500 µl reservoir solution in each well. The crystal of ligand bound form was obtained with a reservoir solution containing 0.1 M sodium acetate trihydrate buffer (pH 4.6), 5 mM resorcinol, and 2 M sodium chloride after a crystallization screen. Selenomethionyl derivative was expressed in *E. coli* B834 BL21(DE3) cells grown in M9 minimum medium supplemented with 50 mg/L L-selenomethionine and then purified and crystallized as described for native protein. The ligand free crystals were obtained in solution containing 3.0 M ammonium sulfate and 0.1 M Tris buffer, pH 8.0.

### Data collection and processing

The crystals of RolR used in data collection were dipped into cryo-protectants (15% PEG3350, 0.2 M ammonium sulphate, 0.1 M sodium acetate buffer, pH 5.0, and 15% glycol) for about 15 seconds after mounted in nylon cryoloops (Hampton Research) and then flash cooled in the stream of liquid nitrogen at 95 K. Multiwavelength anomalous diffraction data sets of ligand bound form were collected from a selenomethionyl crystal at 0.9789 Å, 0.9794 Å, and 0.9500 Å, respectively, at beam line 5A of KEK, Photon Factory, Japan. The data of ligand free crystal were also collected at beam line 5A of KEK with wavelength 1.0000 Å. All the data frames were processed with the program package MOSFLM [20]. The statistics of the data collection was summarized in Table 1.

### Structure determination and refinement

The ligand bound structure of RolR was determined using a three-wavelength MAD method due to no signally similar sequence over full length with known structure protein. The program SOLVE was used to determine and refine the positions of the selenium atoms, and the program RESOLVE was then used to perform solvent flattening and initial phase calculations [21], [22]. The initial electron density map was of excellent quality and most sidechains were clearly identifiable. Automatic model building was performed with the program ARP/wARP [23]. Most of the residues were automatically built into the density map and the remaining residues were manually built using the graphics package O [24], followed by the structural refinement using the program CNS [25]. Five percent of the reflections were randomly chosen for free R calculations and were excluded from the refinement [26]. At last, the stereochemical assessments of the structure were performed by PROCHECK [27]. The ligand free structure was determined by molecular replacement method using N- and C-domains of the ligand bound RolR as the molecular probe, respectively. And the following refinement was the same as that of the ligand bound form. The figures were prepared by Molscript [28] and Pymol [29].

### Site-directed mutagenesis

Genes of mutant D94A, R145A, R148A and D149A were constructed using PCR methods and then confirmed by DNA sequencing. The mutant proteins were expressed and purified following the method described for the recombinant protein RolR. The mutant proteins' performances in expression and purification are similar to that of wild-type protein. In contrast, the CD spectrum of these proteins show all of them fold as native form (Figure S2) and the mutations do not disturb protein folding.

### Isothermal titration calorimetry assay

The calorimetric constants of RolR and mutant proteins binding ligand were determined using a ITC200 isothermal titration calorimeter (200 µL cell, Microcal, Northampton, MA). The concentrations of proteins and ligand are 0.07–0.10 mM and 2–5 mM. The titrations were performed at 25°C with stirring at 1000 rpm and consisted of 24 injections of 1.3 µL separated by 250 s. The binding constants were calculated using Origin provided by ITC200.

### Protein Data Bank accession number

Coordinates and structure factors for the structure of RolR without and with the resorcinol have been deposited at the Protein Data Bank with accession numbers 3AQS and 3AQT.

## Supporting Information

Figure S1 Effects of resorcinol on the binding affinity of RolR to intergenic DNA sequence between *ncgl1110* and *ncgl1111* (From Huang Y. (2007) Genetic Characterization of the Resorcinol Catabolic Pathway and the Transcriptional Regulator for this pathway in *Corynebacterium glutamicum*. Doctoral Thesis, Chinese Academy of Science [10]). 0.1 pM DNA fragment (DNA sequence: 5′-AGGGAAAACC TTAGCTGATC TGCGGTGACT TAAATATAAG GGGGTGGAAT GGGGGTATTG TAAAATCTGA ACCCTTGTTC ATTTATGAAT CATGATTCAG AATGTGATCT AGATAATGTT GTTCAGTTCA CTATTCAAGA AGGGTTAGAT CCC-3′) and 1 pM RolR were added. The resorcinol was added to a final concentration of 1 mM.
(TIF)

Figure S2 CD spectra of the wild-type and the mutant proteins of RolR. Purified protein (0.4 ml of 0.3 mg.ml^−1^) in 50 mM PBS buffer (pH 8.0) was determined with wavelength ranged from 200 to 260 nm using a Jasco J-8100 CD spectrometer.
(TIF)
