# Supplementary figures and images for: Crystal Structures of the Transcriptional Repressor RolR Reveals a Novel Recognition Mechanism between Inducer and Regulator
Source: PLoS One. 2011 May 3;6(5):e19529. doi: 10.1371/journal.pone.0019529 (PMC3086911; doi:10.1371/journal.pone.0019529)

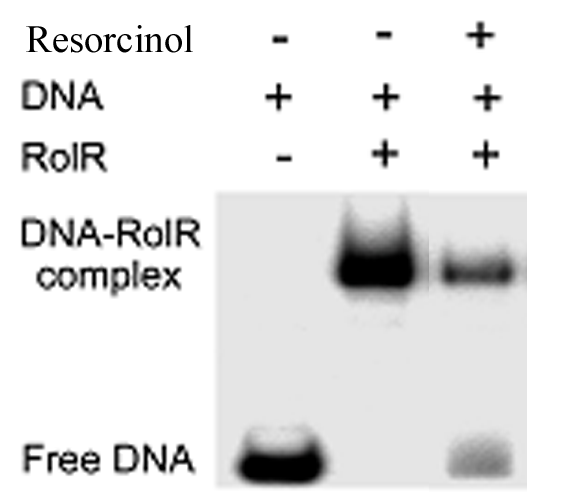

Supplement: Figure S1 — Effects of resorcinol on the binding affinity of RolR to intergenic DNA sequence between ncgl1110 and ncgl1111 (From Huang Y. (2007) Genetic Characterization of the Resorcinol Catabolic Pathway and the Transcriptional Regulator for this pathway in Corynebacterium glutamicum. Doctoral Thesis, Chinese Academy of Science [10]). 0.1 pM DNA fragment (DNA sequence: 5′-AGGGAAAACC TTAGCTGATC TGCGGTGACT TAAATATAAG GGGGTGGAAT GGGGGTATTG TAAAATCTGA ACCCTTGTTC ATTTATGAAT CATGATTCAG AATGTGATCT AGATAATGTT GTTCAGTTCA CTATTCAAGA AGGGTTAGAT CCC-3′) and 1 pM RolR were added. The resorcinol was added to a final concentration of 1 mM. (TIF) [file pone.0019529.s001.tif]

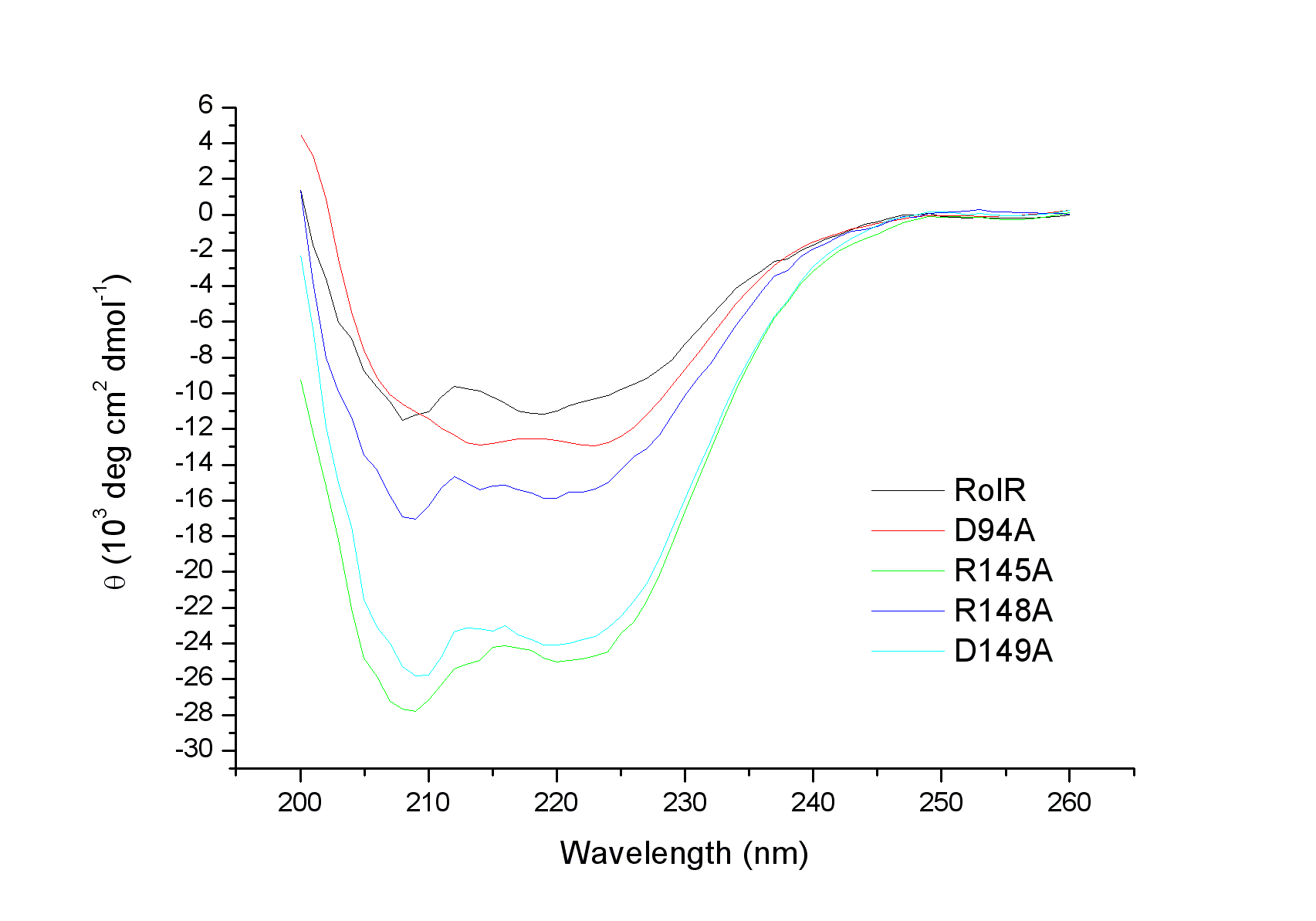

Supplement: Figure S2 — CD spectra of the wild-type and the mutant proteins of RolR. Purified protein (0.4 ml of 0.3 mg.ml−1) in 50 mM PBS buffer (pH 8.0) was determined with wavelength ranged from 200 to 260 nm using a Jasco J-8100 CD spectrometer. (TIF) [file pone.0019529.s002.tif]
